# Clinical Remarks upon Surgical Cases in the Buffalo General Hospital

**Published:** 1867-11

**Authors:** J. F. Miner


ART. II—*Clinical Remarks upon Surgical Cases in the Buffalo General Hospital.* By J. F. Miner, M, D.

Gentlemen:—I have first to present you some cases, showing the results of operations made by my predecessor in the hospital service, Prof. Eastman. Those of you who attended the preliminary term will remember them; they were made in your presence.

The first was the removal of a scirrhus breast. The tumor was small, but unmistakable in character. It was removed in connection with the mammary gland, which was involved in the disease, and which was also small. You will observe that in two and a half weeks' time the wound is nearly healed and the patient will soon be discharged.

The question above all others in importance concerning it is, will it return? It was a favorable case for removal, and it was beyond all doubt the safest and best, indeed the only rational plan of procedure; but it *is liable to return.* At the present time, some of the best pathologists in the world believe that there is a time in the early history of malignant disease when it is purely local, and when if removed there is no general infection or impregnation of the general system. Others regard the appearance of such growths as local evidence of a constitutional bias, and that the system must first be diseased before such local tumors can make their appearance. Without stopping to discuss these questions farther than to indicate my belief, that the former seems to my mind sustained by the best evidence, I will close by saying, that all malignant tumors, in their early stages, should be, if possible, removed; it offers the safest and most satisfactory plan of treatment yet proposed. They are liable to return; if certainly malignant in character they will sooner or later make their re-appearance and often should be again and again removed. But there comes a time when they should be no longer interfered with; when the glands in the neighborhood become enlarged and show absorption of the poisonous material; when the general system becomes debilitated, and when to again disturb the local disease is only to sooner hasten the fatal result. Briefly stated then, malignant tumors, when favorably situated, should be removed in their early stages; they will return, but this does not change the propriety and necessity of early operation. A time comes in the progress of such disease when removal is no longer possible or desirable, when it will not do any good, will, indeed, do harm.

The second case to which I will direct your attention was also operated upon by Prof. Eastman. The patient has suffered for a long time with perineal fistulæ, and has been in the hospital several months. When he was first examined here the stricture of the urethra was very close. It has been gradually dilated by the careful introduction of catheters and other dilating instruments until the largest size could be readily introduced into the bladder. It has also been obvious for some time, that the urethra contained calculi or fragments of calculi; no stone could be detected in the bladder. Various efforts had been made to extract these fragments, but all with but partial success, small bits only being removed. The operation, which many of you witnessed, consisted in opening down upon a grooved director, much as in the operation of removing stone from the bladder, the incision was carried along sufficiently to expose the stone and it was grasped by forceps and removed. It would have done itself credit, so far as size is concerned, had it have been found in the bladder itself; it would measure an inch in its long diameter and three-fourths of an inch in its short diameter; was smooth in outline and very much resembled a stone from the bladder. The probabilities are that the greater part of its growth was attained while situated in the urethra, and it also appears probable that it has been the main cause of all the suffering of the patient. He is not yet recovered. but seems to be doing as well as the nature of the case could permit.

For our third and last patient this morning I will give you opportunity to witness the dressing of a recent case of transverse fracture of the patella. The patient's shoulders are elevated, and the leg is laid upon a well padded splint, and also elevated, thus causing relaxation of the recti muscles which are inserted into the upper margin of the patella.

The fragments are now approximated, as near as possible, though the upper fragment has been drawn five or six inches from the lower; adhesive plaster is applied so as to retain them as near in apposition as may be, and over all a bandage is carefully applied so as to steady the fragments aud approximate them, if possible, more closely.

The best result attainable in such a case is not satisfactory. It is probable that the union will not be by bone, but that a ligament will at length be formed, connecting the fragments. The leg will then be useful, and must be accepted by its owner as the best for him which he can have.

This is an important and interesting case, and if any of you should ever meet a similar one in private practice, do not promise anything but an imperfect and unsatisfactory result. There are many instructive cases now in the wards, but our time has been too much occupied in dressings and other manipulations for detailed description this morning. You shall hereafter have ample opportunity of examining them and learning what treatment is adopted.

